# Impact of Second Opinions on Time to Treatment of Breast Cancer

**DOI:** 10.1245/s10434-025-17934-1

**Published:** 2025-08-02

**Authors:** Pooja M. Varman, Andrew Conner, William C. Bennett, David Taft, Anna M. Chichura, Vincent Wu, Paula Escobar, Kimberly P. Woo, Amanda Cech, Christine Rosenberg, Zahraa Al-Hilli

**Affiliations:** 1 Department of General Surgery, Digestive Disease Institute, Cleveland Clinic, Cleveland, OH USA; 2 Department of Diagnostic Radiology, Imaging Institute, Cleveland Clinic, Cleveland, OH USA; 3 Division of Surgical Oncology, Department of Surgery, University of Illinois Cancer Center, Chicago, IL USA

**Keywords:** Breast cancer, Second opinion, Time to treatment, Surgical oncology, External diagnosis, Diagnostic workup, Multidisciplinary care

## Abstract

**Background:**

The Commission on Cancer (CoC) advocates that upfront breast surgery is performed within 60 days of diagnosis of stage I–III breast cancer, but it is unknown whether patients seeking a second opinion at an outside institution experience increased delays in time to first treatment (TTT). This study compares TTT between externally diagnosed patients (EDP) and internally diagnosed patients (IDP) with breast cancer.

**Methods:**

This retrospective cohort study included patients with stage 0–III breast cancer treated at a single institution between January and July 2024. Patient demographics, date of multidisciplinary consultations, number of additional imaging tests and biopsies performed, and treatment information were reviewed. Two different TTT were calculated: time from biopsy to treatment and time from first surgical oncology clinic appointment at our institution to treatment (TCT).

**Results:**

A total of 226 patients (113 IDP, 113 EDP) were included. Median TCT was 21 days (IQR 12, 29 days) with no difference between EDP and IDP (20 vs. 21 days, *p* = 0.6594). EDP required additional imaging and biopsies more frequently than IDP (88.5% vs. 65.5%, *p* < 0.001). Additional workup and breast surgery with concurrent reconstruction increased median TCT (11 vs. 22 days, *p* < 0.0001, and 20 vs. 26 days, *p* = 0.0007, respectively).

**Conclusions:**

Obtaining a second opinion increased the overall time from diagnosis to treatment but remained within the CoC standard. There was no difference in TCT between EDP and IDP once initiating care at our institution. Patients should not be discouraged from obtaining a second opinion on the basis of concerns about time to treatment.

Timeliness is a critical dimension of high-quality breast cancer care, as delays can adversely affect overall survival, disease-specific survival, and breast cancer-specific mortality.^1,2^ In recognition of this, the Commission on Cancer (CoC) recommends therapeutic breast surgery in the non-adjuvant setting be performed within 60 days of diagnosis for stage I–III breast cancer.^3^

Meeting this benchmark can be challenging due to the inherent complexities of managing breast cancer and coordinating multidisciplinary care. Pretreatment evaluations such as additional imaging, biopsies, and consultations often extend treatment timelines.^4^ One common contributor to these delays is the pursuit of a second opinion.

Second opinions are frequently sought by patients to gain more information, certainty about their diagnosis and treatment plan, and trust in their care team.^5^ These evaluations can meaningfully influence clinical management, sometimes leading to the identification of additional malignant lesions, diagnostic changes, and alterations to the treatment plan.^6^ However, they can also introduce delays in time to first treatment (TTT) due to the need for additional care coordination and diagnostic workup.

In this study, we examine the impact of diagnosis location (internal vs. external) on treatment timeliness for newly diagnosed patients with breast cancer at Cleveland Clinic. Our primary aim is to compare time to treatment between patients initially diagnosed externally and those diagnosed internally within our health system, using biopsy date, first surgical consultation, and initiation of treatment as key milestones. Our secondary aim is to assess the extent to which additional diagnostic procedures contribute to treatment delays.

## Methods

Following approval of the Institutional Review Board at Cleveland Clinic, we conducted a single-institution, retrospective cohort study to investigate the impact of external diagnosis and additional diagnostic workup on breast cancer time to treatment.

### Patient Population

A retrospective review of patients with a new breast cancer diagnosis who visited the Main Campus of Cleveland Clinic (CC) in Cleveland, OH for an outpatient surgical consultation between January 2024 and July 2024 was performed. CC is a quaternary hospital with comprehensive cancer care. Multidisciplinary care is coordinated between healthcare professionals in pathology, radiology, surgical oncology, medical oncology, radiation oncology, plastic surgery, and genetics. Patients were identified by the specific visit type “New Breast Cancer” associated with their clinic appointment in the electronic medical record (EMR). All patients with this visit type have a new, biopsy-proven diagnosis of stage 0–III breast cancer. Two groups were compared: (1) those who completed their initial workup and received a diagnosis of breast cancer at CC (internally diagnosed patients [IDP]) and (2) those who were diagnosed externally and sought a consultation resulting in first treatment at our institution (externally diagnosed patients [EDP]). Externally diagnosed patients included patients with a documented surgical consultation at an outside institution prior to being seen at CC and those who were self-referred. Exclusion criteria were (1) metastatic disease, (2) initiation of first treatment outside our institution, and (3) patient choice to decline standard of care, which was determined by documentation of such and/or absence of scheduled treatments at our institution.

### Data Collection

Information on patient demographics, clinical staging, dates of key medical encounters, additional imaging and biopsies, and treatment modality were retrieved for each patient. Patient demographics included date of birth, age at diagnosis, sex, race/ethnicity, zip code, city, state, and location of breast cancer diagnosis. Clinical and pathologic staging of their breast cancer were noted. Additional workup was defined as any further imaging that was recommended by a radiologist and/or surgeon after the initial biopsy. This included mammogram, ultrasound, magnetic resonance imaging (MRI), ultrasound-guided biopsy, stereotactic biopsy, and MRI-guided biopsy. Additional workup recommendations were tracked for all patients; for internal patients, this was based on the initial radiology report, and for external patients, this was based on the overread performed at our institution. Indications for breast MRI were noted. The need for additional biopsies and subsequent biopsy results were also captured. The following dates of key medical encounters were retrieved: biopsy, first surgical consultation at CC, first medical oncology consultation at CC, first radiation oncology consultation at CC, first plastic surgery consultation at CC, first genetics consultation at CC, and first treatment. Finally, we retrieved the first treatment modality, and when surgery was performed, the type of surgery.

### Definitions of Time to Treatment Metrics

Time to treatment (TTT) is a universal outcome measure referring to the interval between initial cancer diagnosis and the start of treatment. In this study, we define time from biopsy to first treatment (TBT) and time from first surgical oncology clinic appointment at our institution to first treatment (TCT). TBT aligns with TTT, whereas TCT captures any treatment delays after initiating care within our system.

### Statistical Analysis

On the basis of our institution’s historical trends in TTT and ratios of internally to externally diagnosed patients, a total of 226 patients (113 patients in each group) were needed to identify meaningful differences in time to treatment metrics between IDP and EDP. A conventional minimum of 80% power was chosen with two-sided type I error rate of 5%. Descriptive statistics were used to summarize baseline characteristics and diagnostics, stratified according to the two patient groups. Categorical variables were compared using chi-squared tests, and ordinal variables were compared using Wilcoxon rank-sum tests. Ordered logistic regression was used to examine the association between diagnostics and time to treatment. All tests were two-sided, and *p* value < 0.05 was considered statistically significant.

## Results

### Study Population

The first 113 patients to meet our eligibility criteria from each group within the designated time period were identified. Table 1 presents the patient and tumor characteristics represented in our participants. The median age of our cohort was 59.8 years. Racial distribution was majority white (*n* = 183, 81.0%) and Black (*n* = 30, 13.3%). Very few IDP (*n* = 7, 6.2%) lived out-of-state, while 38.1% (*n* = 43) of EDP lived out-of-state. The insurance status of our patients with breast cancer at CC aligns with other CoC facilities as of 2022: 47.1% Medicare, 44.6% private insurance, 6.5% Medicaid, and 1.1% uninsured. Most patients had clinical tumor stage 1 (cT1) disease (*n* = 124, 54.9%) and clinical nodal stage 0 (cN0) disease (*n* = 198, 87.6%), with no significant difference in disease complexity between IDP and EDP.

**Table 1:** Demographic, tumor, and treatment characteristics of internally and externally diagnosed patients

Demographic, tumor, and treatment characteristics	All patients *n* = 226 (%)	Internal patients *n* = 113 (%)	External patients *n* = 113 (%)
Mean age at diagnosis [SD]	59.8 [13.0]	61.8 [12.8]	57.3 [12.8]
Female	225 (99.6)	112 (99.1)	113 (100)
Race			
White	183 (81.0)	82 (72.6)	101 (89.4)
Black	30 (13.3)	27 (23.9)	3 (2.7)
Other	13 (5.7)	4 (3.5)	9 (8.0)
Out-of-state	50 (22.1)	7 (6.2)	43 (38.1)
Clinical T			
cT0/is	36 (15.9)	16 (14.2)	20 (17.7)
cT1	124 (54.9)	66 (58.4)	58 (51.3)
cT2	51 (22.6)	22 (19.5)	29 (25.7)
cT3	13 (5.8)	7 (6.2)	6 (5.3)
cT4	2 (0.9)	2 (1.8)	0 (0)
Clinical N			
cN0	198 (87.6)	96 (85.0)	102 (90.3)
cN1	26 (11.5)	16 (14.2)	10 (8.8)
cN2	1 (0.4)	1 (0.9)	0 (0)
cN3	1 (0.4)	0 (0)	1 (0.9)
Pathologic T			
pT0/is	37 (16.4)	20 (17.7)	17 (15.0)
pT1	119 (52.7)	62 (54.9)	57 (50.4)
pT2	46 (20.4)	20 (17.7)	26 (23.0)
pT3	10 (4.4)	6 (5.3)	4 (3.5)
pT4	2 (0.9)	2 (1.8)	0 (0)
Pathologic N			
pN0	130 (57.5)	64 (56.6)	66 (58.4)
pN1	32 (14.2)	16 (14.2)	16 (14.2)
pN2	5 (2.2)	3 (2.7)	2 (1.8)
pN3	4 (1.8)	3 (2.7)	1 (0.9)
pNx	55 (24.3)	27 (23.9)	27 (24.3)
Multidisciplinary consultations prior to treatment			
Surgical oncology	226 (100)	113 (100)	113 (100)
Medical oncology	226 (100)	113 (100)	113 (100)
Radiation oncology	224 (99.1)	112 (99.1)	112 (99.1)
Genetic counseling	139 (61.5)	75 (66.4)	64 (56.6)
Plastic surgery	91 (40.3)	36 (31.9)	55 (48.7)
Initial treatment modality			
Surgery	188 (83.2)	94 (83.2)	94 (83.2)
Neoadjuvant chemotherapy	25 (11.1)	14 (12.4)	11 (9.7)
Radiation therapy	1 (0.4)	1 (0.9)	0 (0)
Endocrine therapy	12 (5.3)	4 (3.5)	8 (7.1)

### Multidisciplinary Consultations

Nearly all patients (*n* = 224, 99.1%) had multidisciplinary visits with breast surgery, medical oncology, and radiation oncology prior to first treatment. Most patients’ (*n* = 182, 80.5%) multidisciplinary consultations were same-day visits. Median time from biopsy to first surgical consultation was 14 days, with IDP seen more quickly (8 days) than EDP (21 days, *p* < 0.0001).

Genetics consultations were arranged for 139 patients (61.5%), including 75 IDP (66.4%) and 64 EDP (56.6%). These visits were not correlated with increased TBT or TCT.

Most patients (*n* = 135, 59.7%) did not require a consultation with plastic and reconstructive surgery (PRS). Of the 91 who did, more EDP (*n* = 55, 48.7%) required consultation than IDP (*n* = 59, 64.8%, *p* = 0.01). Of the patients who had PRS consults, 59 (64.8%) ultimately underwent a combined procedure for breast surgery with reconstruction. The need for PRS consultation and scheduling of a combined reconstructive procedure increased TCT by 6 days (without reconstruction TCT = 20 days, with reconstruction TCT = 26 days, *p* = 0.0007).

### Additional Diagnostic Workup

In this study, 174 (77.0%) patients required additional diagnostic workup with imaging and/or biopsies, as recommended by the surgeon and/or radiologist at our institution. EDP (100, 88.5%) required additional imaging and/or biopsies more frequently than IDP (74, 65.5%, *p* < 0.001). The most common workup needed for IDP was MRI (*n* = 57, 50.4%). EDP required additional mammogram (*n* = 59, 52.2%), ultrasound (*n* = 62, 54.9%), and MRI (*n* = 58, 51.3%) at similar rates. Table 2 presents the need for additional workup in each group.

**Table 2:** Time to treatment outcomes

Time to treatment metric	All patients *n* = 226	Internal patients *n* = 113	External patients *n* = 113	*p* value
Time to first surgical consultation, days	14 (8, 21)	8 (6, 13)	21 (15, 28)	**< 0.0001**
Time to treatment, days				
Median TBT (IQR)	35 (27, 48)	31 (21, 38)	42 (32, 57)	**< 0.00001**
Range of TBT	6–201	6–82	13–201	
Median TCT (IQR)	21 (12, 29)	21 (12, 29)	20 (12, 30)	0.6594
Range of TCT	− 3 to 75^†^	− 3 to 75^†^	0–64	

	All patients *n* = 226	Additional imaging *n* = 174	No additional imaging *n* = 52	*p *value
Median TBT (IQR)	35 (27, 48)	38 (29, 50)	26 (17, 35.5)	**< 0.0001**
Median TCT (IQR)	21 (12, 29)	22 (15, 32)	11 (8, 20)	**< 0.0001**

	All patients *n* = 226	With reconstruction *n* = 68	Without reconstruction *n* = 158	*p* value
Median TBT (IQR)	35 (27, 48)	40 (26, 56)	34 (27, 43)	**0.0063**
Median TCT (IQR)	21 (12, 29)	26 (14, 38)	20 (11, 26)	**0.0007**

Bolded values are significant at α = 0.05

*TBT* time from biopsy to first treatment, *TCT* time from first surgical consultation to first treatment, *IQR* interquartile range

^†^Negative days denotes neoadjuvant endocrine therapy initiation prior to first surgical consultation.

Radiologists recommended additional workup more often for EDP (69.0%) than IDP (26.6%) (*p* < 0.001), while surgeons recommended additional workup with similar frequency in each group (EDP 61.9%, IDP 63.7%, *p* = 0.78). Additional breast MRI was ordered in 50.9% of cases, with no difference in rate of MRI between IDP (*n* = 57, 50.4%) and EDP (*n* = 58, 51.3%). The most common indications for breast MRI were dense breasts, young age, history of breast cancer, invasive lobular carcinoma, to establish baseline prior to NAC, and to evaluate extent of disease and treatment planning. Excluding any additional breast MRI, the need for additional workup was still greater in the EDP (77.9%) than IDP (51.3%). Table 3 presents all additional workup performed in our cohort.

**Table 3:** Additional diagnostic workup recommended and performed for internally and externally diagnosed patients

Additional diagnostic workup	All patients *n* = 226 (%)	Internal patients *n* = 113 (%)	External patients *n* = 113 (%)	*p* value
Recommended	170 (75.2)	74 (65.5)	96 (85.0)	**0.001**
By surgeon	142 (62.8)	72 (63.7)	70 (61.9)	0.783
By radiologist	108 (47.8)	30 (26.6)	78 (69.0)	**< 0.001**
Performed	174 (77.0)	74 (65.5)	100 (88.5)	**< 0.001**
Imaging	171 (75.7)	73 (64.6)	98 (86.7)	**< 0.001**
Mammogram	67 (29.7)	8 (7.1)	59 (52.2)	**< 0.001**
Ultrasound	92 (40.7)	30 (26.6)	62 (54.9)	**< 0.001**
MRI	115 (50.9)	57 (50.4)	58 (51.3)	0.894
Staging CT scans	49 (21.7)	27 (23.9)	22 (19.5)	0.420
Biopsy	66 (29.2)	28 (24.8)	38 (33.6)	0.143
Ultrasound-guided	31 (13.7)	16 (14.2)	15 (13.3)	0.847
Stereotactic	15 (6.6)	5 (4.4)	10 (8.9)	0.182
MRI-guided	27 (12.0)	12 (10.6)	15 (13.3)	0.538

Bolded values are significant at α = 0.05

Additional diagnostic workup ultimately led to additional biopsies in 66 patients (29.2%), of whom 22 patients were diagnosed with an additional site of cancer (9.7% of the cohort, 33.3% diagnostic yield). Of the 74 IDP who underwent additional workup, 28 had an additional biopsy, and 10 were ultimately diagnosed with an additional cancer (13.5% of all IDP with additional workup). Meanwhile, of the 100 EDP who underwent additional workup, 38 had an additional biopsy, and 12 were ultimately diagnosed with an additional cancer (12.0% of all EDP with additional workup). There was no difference in the frequency of need for additional biopsy between IDP (26.6%) and EDP (36.3%). In some cases (5 IDP, 11 EDP), an ipsilateral biopsy was recommended but not performed, as the patient was already planning to undergo ipsilateral or bilateral mastectomy. These patients were tracked as obtaining additional imaging but excluded from the analysis of diagnostic yield because no additional diagnosis was made prior to treatment.

**Fig. 1.**
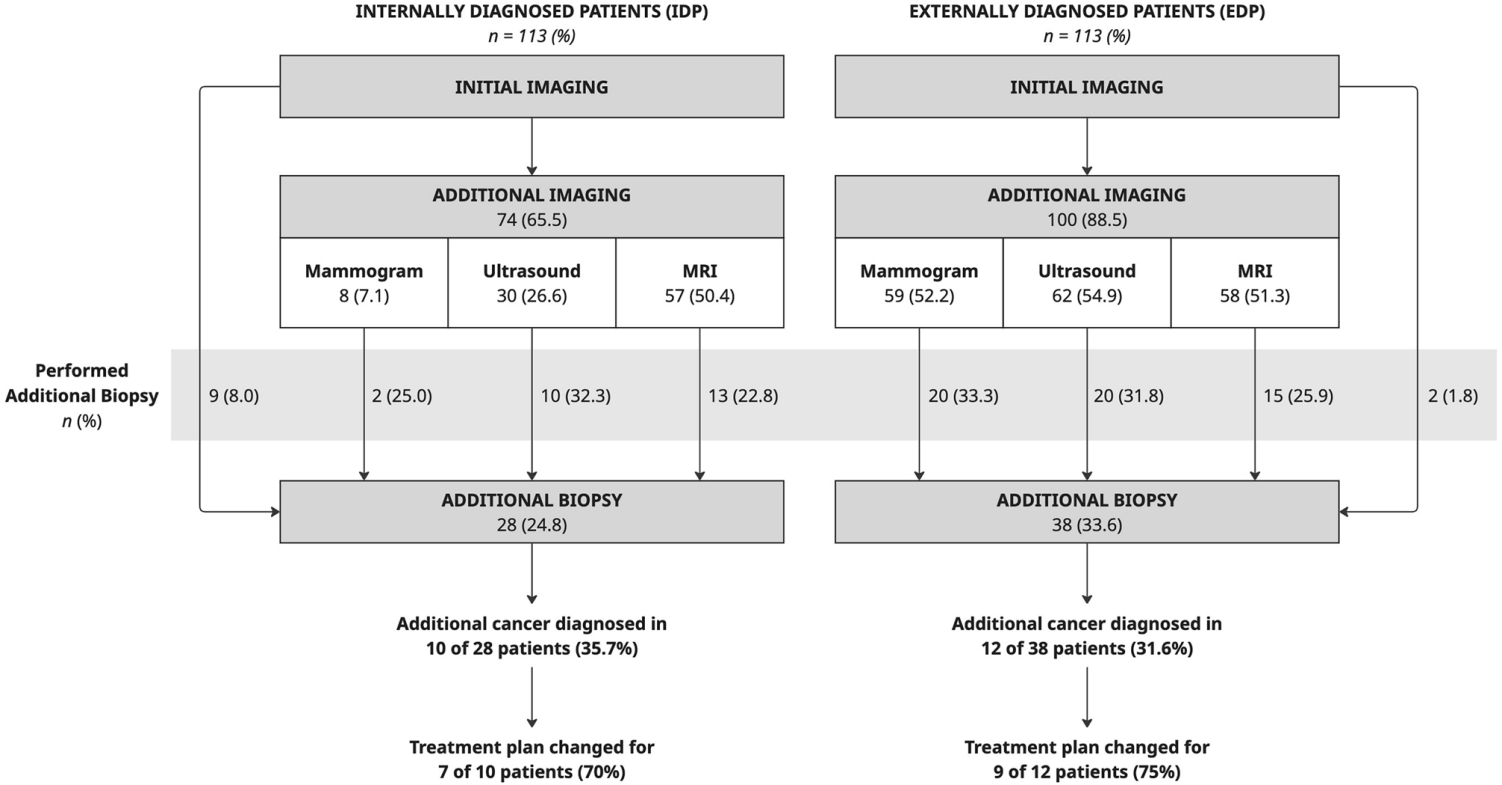
Diagnostic yield of additional workup performed

Diagnostic yield of additional biopsies was similar in both groups: 10 of 28 (35.7%) IDP and 12 of 38 (31.6%) EDP were diagnosed with an additional cancer. Ultimately, treatment plans were affected in 16 of 22 patients diagnosed with a new cancer on the basis of additional biopsy results. These changes included a more extensive ipsilateral procedure (*n* = 9) and addition of a contralateral procedure (*n* = 4). For three patients, surgical planning was intentionally deferred until after additional biopsy. Treatment plan changes were not applicable for the remaining six patients: five patients’ first treatment was neoadjuvant chemotherapy and one patient with an inoperable tumor had radiation therapy. Figure 1 shows the diagnostic yield from additional workup for both groups.

Requiring additional workup—regardless of whether that included another image or biopsy and irrespective of diagnosis location—increased TCT by 12 days (*p* < 0.0001): median TBT for those not requiring additional workup was 26 days (interquartile range [IQR] 17, 35 days) and for those requiring additional workup was 38 days (IQR 29, 51 days). Compared with no additional testing, an additional ultrasound increased TBT by 12 days (*p* < 0.0001), mammogram by 13 days (*p* < 0.0001), and MRI by 9 days (*p* < 0.0001). There was a 10-day delay in TBT (*p* < 0.0001) in patients requiring additional biopsy (43.5 days, IQR 35, 60 days) as compared with those who did not (33.5 days, IQR 22.5, 43 days).

**Fig. 2.**
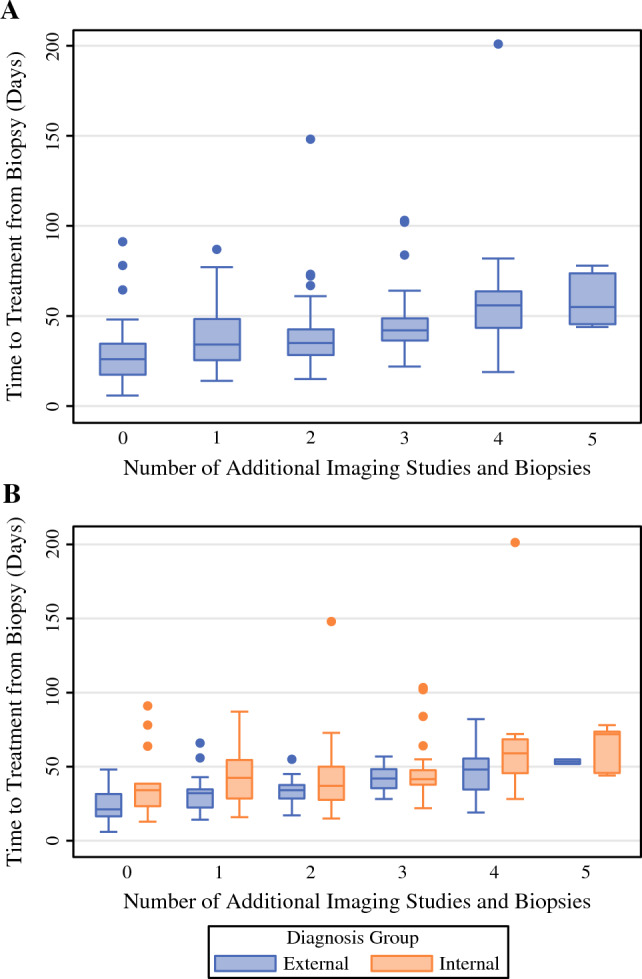
Box plot showing distribution of TBT with each additional diagnostic test; (**A**) for the whole cohort, (**B**) stratified by diagnosis location

Ordered logistic regression was performed to determine the relationship between number of additional tests performed and TBT. We found a coefficient of 0.689 and subsequent odds ratio of 1.99 indicating that for each additional test, the odds of delaying TBT is expected to increase by a factor of 1.99 (*p* < 0.001). Figure 2 depicts the distribution of TBT for increasing number of tests performed.

### Treatment Initiation

**Fig. 3.**
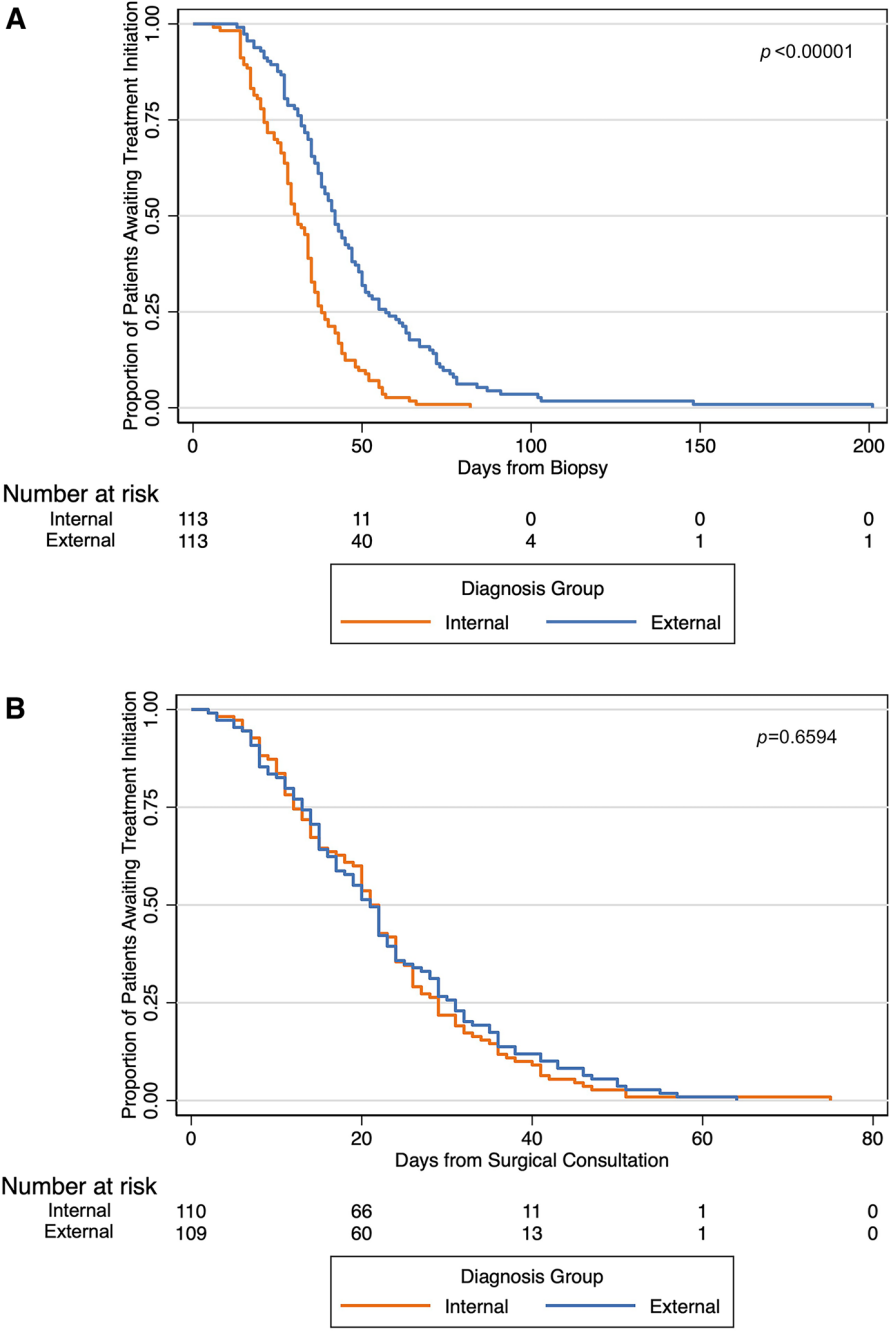
Kaplan–Meier curves of time to treatment, stratified by diagnosis location; (**A**) time from biopsy to treatment, (**B**) time from surgical consultation to treatment

The distribution of initial treatment modality was similar for IDP and EDP (*p* = 0.44). Among IDP, 83.2% underwent surgery first and 12.4% received neoadjuvant chemotherapy. Among EDP, 83.2% had surgery first and 9.7% had neoadjuvant chemotherapy. Median time from biopsy to first treatment (TBT) for the cohort was 35 days (IQR 27, 48 days). Treatment was initiated more quickly for IDP (31 days) than for EDP (42 days), which was a statistically significant difference (*p* < 0.00001). Median time from first surgical oncology clinic appointment at our institution to first treatment (TCT) for the cohort was 21 days (IQR 12, 29 days) with no statistical difference between EDP (20 days) and IDP (21 days, *p* = 0.66). In Fig. 3, Kaplan–Meier curves depict the proportion of patients of awaiting treatment as time progresses, stratified by diagnosis location.

## Discussion

In this retrospective cohort study, we found that patients who sought a second opinion at our comprehensive cancer center after an external diagnosis of stage 0–III breast cancer experienced a 10-day increase from biopsy to treatment (TBT) as compared with those diagnosed internally at our institution. Studies show that transferring care to another center adds between 5 and 9 days to time to treatment, with some reports indicating significantly longer delays.^7,8^ For instance, a nationwide study in Japan found that seeking second opinions for breast cancer delayed the start of treatment by an average of 22 days.^9^ Blazek et al. observed delays of 20–58 days among low-income, Black, and Latina patients seeking second opinions.^10^ However, when we considered time from the first surgical oncology visit to treatment (TCT), there was no difference between internally (IDP) and externally diagnosed patients (EDP). This indicates that most delays in treatment between the two groups occurred between diagnosis and initial consultation, but once they established care within our system, both cohorts embarked on a similar timeline for initiating treatment.

The need for additional workup was the strongest predictor of delay in treatment. EDP were significantly more likely to undergo further imaging and biopsies than IDP, even when excluding MRI, a modality that has been reported to delay time to surgery by 11 days.^11^ As such, the increased TBT among EDP appears to be driven largely by the need for any additional diagnostic workup. Additional testing led to diagnosis of new cancer 12.6% of the time, with no difference between IDP and EDP. These new diagnoses had an impact on treatment plan in 72.7% of cases, again, with no difference between IDP and EDP. Beyond cancer diagnostic yield, additional imaging was useful in further characterizing known lesions and establishing baselines prior to NAC.

Overall, the diagnostic burden was notably higher for EDP than IDP. Surgeons recommended additional workup for EDP and IDP at similar rates, while radiologists were more likely to recommend additional workup in externally diagnosed cases. This could reflect variable imaging quality, incomplete workups, or lack of access to prior imaging. For many patients, the additional recommended workup was performed on or before the date of their initial surgical consultation; others obtained their imaging afterwards and required follow-up appointments prior to surgery. Completing diagnostic workup before scheduling surgical consultation can reduce the need for further follow-up and reduce the time to treatment, so long as the initial surgical touchpoint is not unnecessarily delayed.

Coordinating multidisciplinary care with medical oncology, radiation oncology, plastic surgery, and genetic counseling can also contribute to the time cost of cancer treatment. In our study, nearly all patients were seen by medical oncology and radiation oncology prior to treatment, and most of those appointments were scheduled on the same day as the surgical consultation. Given the small number of patients whose multidisciplinary consultations were not scheduled on the same day, meaningful timeline delays of different-day appointments could not be ascertained. It is important to note that seeing medical oncology and radiation oncology is not a rate limiting factor to proceed with surgery if neoadjuvant treatment is not indicated. Obtaining a plastic surgery consultation and scheduling a combined breast procedure with reconstruction amounted to a 6-day delay. Meanwhile, genetics consultations did not result in a delay. This is consistent with existing literature showing hereditary breast cancer evaluation and genetic testing does not delay time to treatment for patients with breast cancer.^12^

Importantly, the median TBT for both groups remained well within the 60-day benchmark set by the CoC, reinforcing the feasibility of timely treatment initiation even in the context of second opinions, additional workup, and care coordination. This finding has critical implications for patient counseling and institutional operations. While delays in cancer care have been associated with poorer outcomes in some studies, our data suggest that patients pursuing second opinions at comprehensive cancer centers can likely still receive care within nationally accepted timelines. In 2014, our institution initiated a multidisciplinary program to reduce time to treatment for patients with cancer and found a 33% reduction in TTT after 5 years of implementing continuous improvement processes and value-stream mapping.^13^ The absence of a difference in TCT between groups in our study further underscores the efficiency of care once patients are integrated in our institutional workflow. While many hurdles have been overcome with streamlined multidisciplinary coordination, work remains to expedite entry into our system for external patients. Improving image sharing processes, standardizing external workup protocols, and fostering interinstitutional communication may also help reduce the diagnostic burden and expedite care integration for patients referred for second opinions.

Limitations of our study include its retrospective nature and the fact that it was conducted at a single institution, which may limit generalizability. However, we did find our average treatment times to be in accordance with recent national data.^14^ Additionally, some externally diagnosed patients did not have a documented surgical consultation prior to being seen at CC; this would likely have delayed time to treatment further and should be considered in future studies. While we excluded patients with metastatic disease and those treated externally, unmeasured confounders such as patient preferences, complex medical or surgical history, insurance-related delays, and psychosocial factors may have influenced TTT, as shown in other studies.^15–18^ We did not track incidence of upstaging or survival metrics, as this study focused on time to treatment metrics. However, inclusion of these additional clinical outcomes would complement the quality outcomes reported here.

Future directions for this research should consider other sources of delay at various timepoints after initial diagnosis. While many of the processes in place at our institution are likely shared with other comprehensive cancer centers, multi-institutional studies including other comparable centers can demonstrate equity and timeliness of treatment of externally diagnosed patients, eventually aiming to produce generalizable results.

## Conclusions

Our study supports that although externally diagnosed patients experience modest delays in time to treatment compared with internally diagnosed patients, these delays are not clinically significant and should not deter patients from seeking second opinions. Additional diagnostic workup is a major contributor to delays and must be expedited by institutions. Institutions should focus on reducing logistical barriers and streamlining care pathways to ensure timely access to the system and equitable multidisciplinary cancer care.
